# An evaluation of the Compassion, Inclusion, and Engagement initiative: learning from PWLE and communities across British Columbia

**DOI:** 10.1186/s12954-023-00819-5

**Published:** 2023-07-14

**Authors:** Sally Maguet, Nancy Laliberte, Laura Moore, Tammy Milkovich, Charlene Burmeister, Marnie Scow, Wendy Sproule, Naomi Dove, Sheila Martens

**Affiliations:** 1 Provincial Health Services Authority, Vancouver, Canada; 2 University of British Columbia, Vancouver, Canada; 3 BC Centre for Disease Control, Vancouver, Canada; 4 Unlocking the Gates, Maple Ridge, Canada

**Keywords:** Compassion, Inclusion, Engagement, PWLE, British Columbia, Developmental evaluation, Harm reduction

## Abstract

**Background:**

The Compassion, Inclusion and Engagement initiative (CIE) was a social contact intervention that operated in British Columbia between 2015 and 2021. The primary objective of CIE was to increase the participation of people with lived experience of substance use (PWLE) in the planning, design, implementation, and evaluation of harm reduction supports and services.

**Case presentation:**

CIE used the developmental evaluation methodology outcome mapping to define and measure progress towards its goals. Developmental evaluation emphasizes learning in contrast to other forms of evaluation which are often more focused on determining the value or success of a project or programme based on predetermined criteria. Outcome mapping is a relational practice which acknowledges that change is achieved by an initiative’s partners and the role of the initiative is to provide access to resources, ideas and opportunities that can facilitate and support change.

**Conclusions:**

Through the implementation and evaluation of CIE, it became clear that directly supporting PWLE facilitated more meaningful and lasting change than solely working to improve the health and social services that supported them. The impacts of the CIE initiative extend far beyond the outcomes of any of the dialogues it facilitated and are largely the result of an increase in social capital. CIE engagements created the opportunity for change by inviting people most affected by the toxic drug supply together with those committed to supporting them, but their ability to bring about systemic change was limited. Both PWLE and service providers noted the lack of support to attend CIE engagements, lack of support for actions that came from those engagements, and lack of PWLE inclusion in decision-making by health authorities as limiting factors for systemic change. The lack of response at a systemic level often resulted in PWLE carrying the burden of responding to toxic drug poisonings, often without resources, support, or compensation.

## Background

A public health emergency was declared in British Columbia (BC) on April 13, 2016, in response to alarmingly high rates of death due to illicit drug overdose. Between January 2016 and December 2020, 6745 people died in BC due to illicit drug toxicity at an average rate of 27 people per 100 000 a year [1]. In 2021 BC lost a shocking 2224 people at a rate of 42.8 people per 100 000 [1]. Illicitl drug toxicity was the leading cause of death for people between 19 and 39 in BC between March 2020 and October 2021, causing more loss of life than heart diseases, stroke, diabetes, or COVID-19 across all age groups [2].

British Columbia is using various strategies to address the emergency including the rapid distribution of Naloxone, the expansion of safe consumption and overdose prevention sites, increasing access to opioid agonist therapies [3, 4], and decriminalization of small amounts of illicit drugs for personal use [5]. It is estimated that harm reduction interventions averted 3030 deaths between April 2016 and December 2017 in BC [6].

There is a growing body of evidence supporting the use of harm reduction strategies for the pragmatic purposes of saving lives and preventing disease [7–9]. Harm reduction can also be justified as a means of relieving human suffering through acts of compassion [10], as a tool of social justice [11], a mechanism to address inequities and an effective means of countering stigmatizing beliefs about PWLE [12]. Harm reduction has been endorsed by the Canadian Nurses Association [13], Doctors of BC [14], and the Canadian Public Health Association [15].

The Compassion, Inclusion and Engagement initiative (CIE) was a collaboration between the First Nations Health Authority (FNHA) and the BC Centre for Disease Control (BCCDC) that operated in BC between 2015 and 2021. The FNHA and the Provincial Health Services Authority, which houses the BCCDC, are the two provincial health authorities that support and collaborate with the provinces five geographically defined regional health authorities. The primary objective of CIE was to increase the participation of people with lived experience of substance use (PWLE) in the planning, design, implementation, and evaluation of harm reduction supports and services across the province. CIE was based on the core principles of harm reduction, equity, social inclusion, and Indigenous cultural safety, which are embodied in the three tenets of the initiative: Compassion, Inclusion and Engagement.

### Compassion

Compassion describes the ability to understand, and desire to alleviate the suffering of others. It is sometimes conflated with pity, empathy, or sympathy, but these terms fail to acknowledge the agency that is conveyed through the concept of compassion and lack an orientation towards action [16].

Compassion is a concept that is embedded in medical codes of ethics [17, 18] and recognized as a key component of effective care [19]. Healthcare practitioner training and structural issues within the healthcare system can result in a reduced capacity for compassion [20, 21]. Compassion fatigue resulting from repeated vicarious trauma and the need for care exceeding a provider’s ability to provide it has gained attention because of the COVID-19 pandemic [22]. When associated with illicit drug overdoses, compassion fatigue within a community can erode support for evidence-based harm reduction services and increase stigma against PWLE [23].

### Inclusion

PWLE are often included in efforts to disseminate information within their communities [24], support the uptake of harm reduction practices [25] and contribute to research projects as research assistants [26, 27]. Peer support workers are also often employed at harm reduction sites and for community outreach [28, 29]. It is less common, however, to include PWLE as advisors or decision makers in the design and planning of harm reduction services [30, 31]. PWLE advocates have been calling for greater PWLE inclusion in decision making and service provision for many years. A national gathering of PWLE organizations in 2014 identified respectful partnerships with service providers and regulators where allies play supportive but not leadership roles as key facilitators for meaningful PWLE participation in the design and delivery of harm reduction services [32]. When included in evaluations of harm reduction or other health services, PWLE are generally only considered the clients, patients, or consumers [33]. The indicators of programme success are rarely defined by PWLE, as they are generally only consulted about outcomes and impacts defined by providers, researchers, or administrators.

PWLE experience social exclusion, discrimination, stigma, and even open hostility in their communities, in their interactions with police and when trying to access healthcare services [34–37]. PWLE can experience stigma even when accessing harm reduction services intended specifically for them [38]. The prohibition of some drugs in Canada and around the world has often led to the social exclusion of PWLE and diminished their participation in social and political structures where decisions are made that affect their lives [39]. Inclusion health is a relatively new concept that seeks to address and prevent health and social inequities among people experiencing poverty, marginalisation, and intersecting health issues [40]. Taking an inclusion health approach acknowledges inclusive processes as part, or even the primary purpose of an intervention. Facilitating inclusive processes and building capacity for those processes to continue was as important to CIE as the more tangible outcomes that immediately resulted from them.

### Engagement

Indigenous PWLE can experience social exclusion even more acutely. Settler colonialism, informed by white supremacy and perpetuated by settler privilege [41], has socially excluded Indigenous people in BC since the late eighteenth century. The social privilege of settlers in BC is the result of centuries of inherent benefits and protections that have not been granted to First Nations and Indigenous Peoples [42]. In 2020, First Nations People died as a result of the toxic drug supply at more than five times the rate of other BC residents and First Nations women died at nearly ten times the rate of other female BC residents [43].

Engagement in health care often refers to collaborative clinical decision-making with patients but can also mean involvement in service development and delivery, policy, and strategic planning [44]. BC’s Ministry of Health’s 2018 Patient, Family, Caregiver and Public Engagement Framework [45] includes three domains of engagement: individual care, bringing in community and system redesign. CIE supported engagement primarily at the community level, although changes in service design, delivery, and the inclusion of PWLE often required the development of enabling policies and advocacy for harm reduction as a strategic priority at the health systems level.

Engagement can occur at many levels. BC’s public engagement framework is based on the International Association for Public Participation’s (IAP2) spectrum of engagement [46] which includes five levels: informing, consulting, involving, collaborating, and empowering. Different levels of engagement require different processes and can lead to very different outcomes. A 2017 review of the spectrum by IAP2 practitioners found that, though it is seen as a valuable tool, the spectrum still centres power with the convener or decision maker who then can choose to transfer power to participants and suggests a second framework that supports participants to lead the processes [47]. CIE intentionally centred PWLE in its facilitated dialogues, planning and evaluation. The use of developmental evaluation methodologies and outcome mapping in particular was well suited for the initiative because it intentionally decentres the influence of the evaluator, allowing the initiative’s partners to define its success.

## Case presentation

CIE was a social contact intervention [48] aimed at increasing the uptake of harm reduction philosophies and principles by service providers and the inclusion of PWLE in harm reduction planning, implementation, and evaluation. It was not a singular curriculum, but an intentional process designed around the principles of equity, and social inclusion. The process included capacity building sessions with PWLE and service providers followed by a collaborative dialogue. The content and desired outcomes of each engagement were co-created with partners in the regional health authorities, local PWLE groups and drug user organizations, community services and Indigenous communities. As CIE became more established, the planning and consultation processes became more inclusive and were often driven by PWLE. CIE also provided small community-based grants to establish and maintain new and emerging PWLE groups.

Evaluation was embedded in the initiative from the beginning. CIE chose to adopt a developmental evaluation (DE) approach. DE is well suited for initiatives that are innovative, early in their development, and approaching complex issues with little agreement on solutions among stakeholders [49, 50]. DE emphasizes learning in contrast to other forms of evaluation which are often more focused on determining the value or success of a project or programme [51]. Unlike other evaluation approaches, DE evaluators are embedded within the project team and actively participate in planning and development of the project [52–54].

CIE used outcome mapping (OM) as the basis for the evaluation. OM is a DE methodology that centres an initiatives’ partners in defining the desired outcomes and ultimate learning of the project or programme. It focuses on the contribution of an initiative to an outcome rather than the attribution of an outcome to the initiative itself. It is a relational practice which acknowledges that change is achieved by an initiative’s partners and the role of the initiative is to provide access to resources, ideas and opportunities that can facilitate and support change [55].

Boundary partners in the OM methodology are individuals or groups that the initiative works closely with, who will be the change agents in their communities. Communities can be defined in terms of geography, social connections, professional networks, communities of practice, or spheres of influence. CIE’s primary boundary partners were PWLE, and health and social service providers. Harm reduction co-ordinators within the regional health authorities were instrumental in providing CIE access to frontline service providers and connecting CIE to harm reduction champions.

Over the course of two years, CIE worked with its boundary partners to define a set of progress markers (PM). PM in the OM methodology describe changes in relationships, attitudes, actions, and behaviours that would bring boundary partners closer to the initiative’s vision. It is important that PM be drafted collaboratively with boundary partners because they understand what the vision would look like in the context of their community. One of the defining characteristics of OM is that it recognizes the importance of an initiative aligning efforts with boundary partners priorities rather than compelling or coercing boundary partners to align with the goals of the initiative. The flexible, participatory, and adaptable nature of PM were particularly well suited to CIE because of the variability between regional health authorities, unique nature of each community and inclusive development process.

**Fig. 1:**
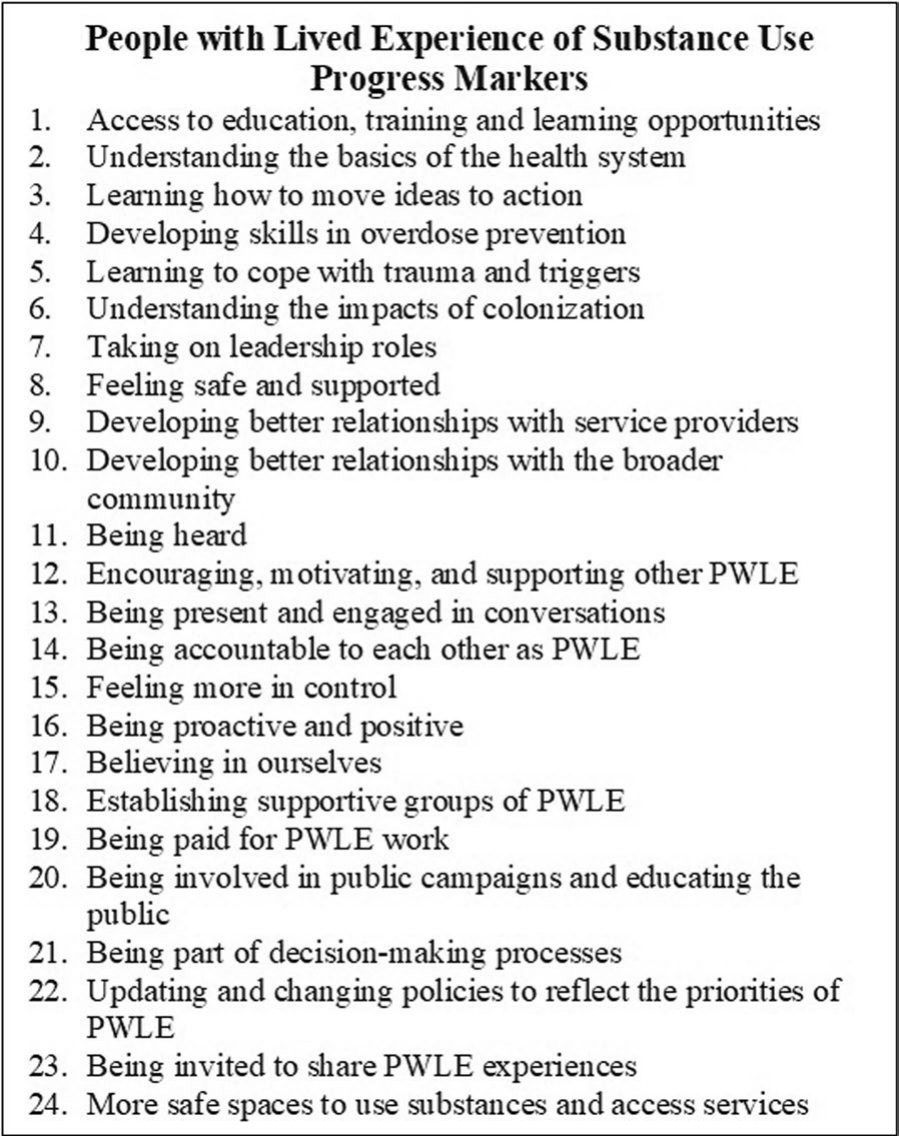
Progress Markers for PWLE

**Fig. 2:**
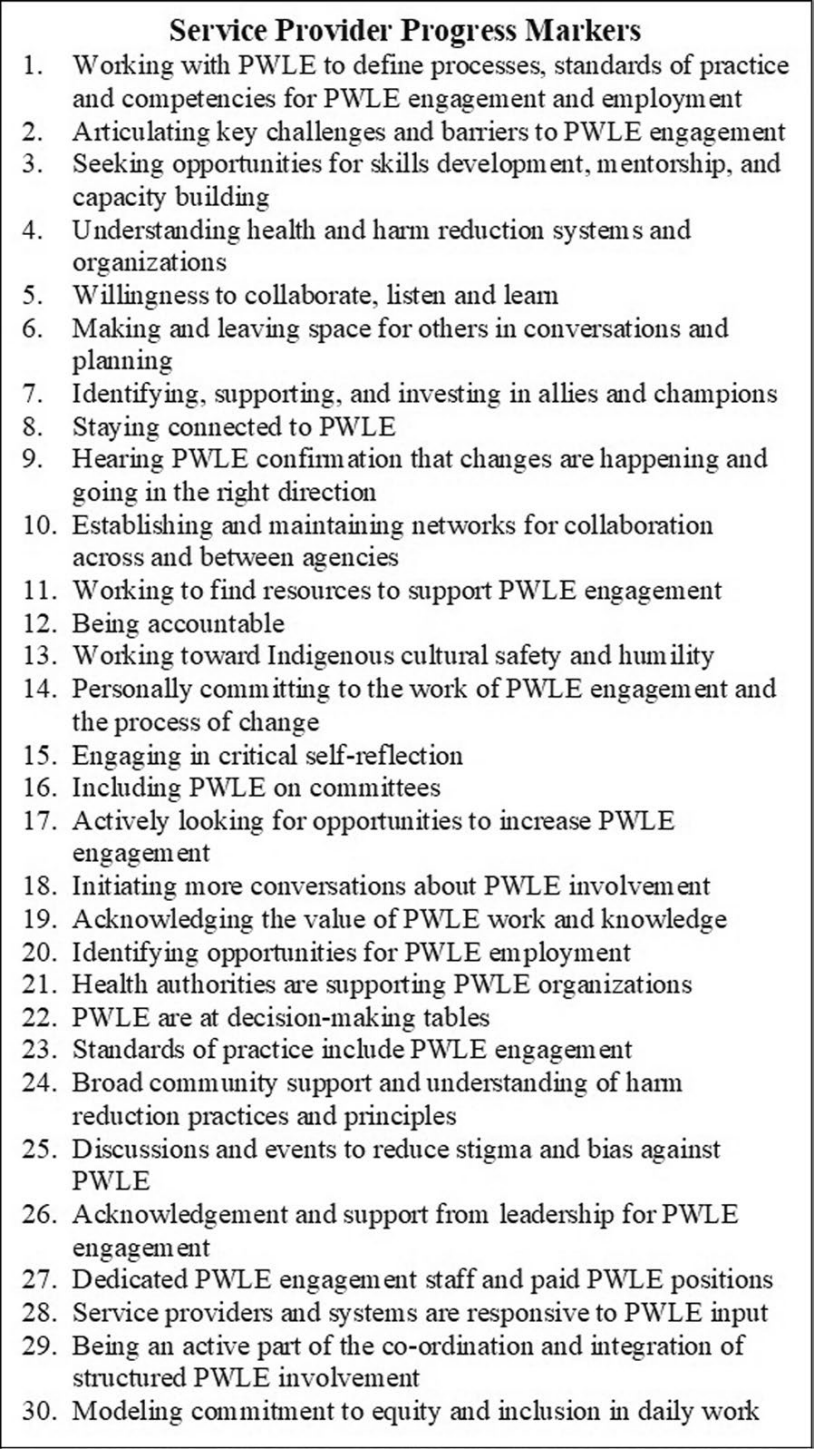
Progress Markers for Service Providers

During the second or third visit to a community, the CIE team worked with PWLE and service providers separately to understand what it would look like if the goals of CIE were brought to fruition in their community. CIE was able to gather responses from 110 PWLE and 130 service providers from 13 communities across the province. Members of the CIE team themed and consolidated the responses from PWLE and service providers separately, resulting in 24 PWLE PM (Fig. 1) and 30 service provider PM (Fig. 2).

### Data collection

Evaluation data were collected throughout the six-year initiative through participant feedback, meeting notes and ad hoc, informal interviews that informed the planning, development and implementation of CIE. CIE received ethics approval from the Community Research Ethics Office for the last phase of its evaluation in December 2020. All 255 PWLE and 206 service providers who had participated in the CIE initiative were invited to participate in an online survey and semi-structured interview in December 2020. A PWLE and service provider specific survey link was sent to all CIE participants who could be reached via email along with an invitation to participate in an interview. PWLE who were not able to receive emails or who were more accessible through phone or social media were contacted through the most appropriate means. CIE recognized that connecting with PWLE through these means was not as equitable as meeting in person in their own communities and may have created barriers to their participation. Those that did not have a current email address and were reached through other means, were also invited to participate in the online survey and offered support to access the internet whenever possible. Only twenty-eight people responded to the online surveys. Given the low response rate, the survey results were not used to inform the findings of the CIE evaluation and did not inform the analysis or conclusions that follow.

Forty-three semi-structured telephone interviews were conducted between December 2020 and March 2021 with respondents from 17 of the 20 communities CIE had worked in. This sample represents approximately 10% of CIE dialogue participants. PWLE were compensated $25/hr for their time if they chose to take the survey, participate in an interview or both. CIE had hoped to integrate data gathering into its engagement activities by conducting in person interviews and focus groups while in communities in 2021. COVID-19 severely restricted CIE’s ability to travel and made in person interviews and data collection unsafe and impractical.

## Analysis

CIE team members used the PM as an analytical framework to code the transcript data, PWLE PM were used to code PWLE transcripts and service provider PM were used to code service provider transcripts. An external analysis team, including PWLE and an Indigenous researcher, was created in December 2020 to provide additional analytical perspectives. The external analysis team used an inductive method, with patterns discerned from interview transcripts used to make inferences about the experience of CIE participants. The analyses of the embedded evaluator and external analysis team were then compared.

### PWLE—progress markers

The PM that appeared most often in the transcripts of PWLE were one, 11, 12, 15, 17, and 18.

PWLE accessed education, training and learning opportunities through CIE sessions as well as workshops provided by PWLE groups using grant funds. Respondents commented most frequently about increasing their confidence in communicating and collaborating with service providers and community leaders. This came primarily through learning about key concepts such as equity, non-violent communication, harm reduction and articulating the meaning of PWLE and PWLE work. The external analysis found similar themes with PWLE reporting the success of peer groups depending partly on access to funding to be able to provide and attend training and employment programmes, improving their skills and enabling them to gain paid employment. One PWLE commented

I learned … that there was a gap between people who hadn’t done drugs and I had done drugs. Trying to bridge that gap between the people I worked with that didn’t come with that experience, helped me to understand that they didn’t understand me, and also I had to learn from you guys that it takes work to become inclusive and to keep trying to collaborate with people. PWLE, Fraser region

The most salient PM was being heard both from the evaluation transcripts and through CIE’s rapid evaluation cycles after each engagement. It was often connected with the idea of feeling safe and supported and developing the skills to effectively communicate and collaborate. The validation that came from being heard was transformative in some cases and made PWLE feel fully human, spurred some to find employment and helping to create and nurture new connections and collaborations with other PWLE, service providers and the broader community. The external thematic analysis found that experiences of speaking up in CIE sessions and at community action tables boosted self-confidence and improved relationships between service providers and PWLE as well as between PWLE groups.

One PWLE commented about the CIE engagement

“It means a lot to us. It was nice to be involved in. People picked up off the ground and people realizing no matter what they’re human, they still have a voice and just to see lights go back in their eyes…it means the world, it meant the world”. PWLE, Northern Region.

Both analyses found that being heard did not always evolve into meaningful inclusion. Some PWLE were not recognized for their contributions or felt their presence was no more than tokenism when no action came from their ideas, or they were not recognized for their contributions. There were also communities where PWLE inclusion did not improve. One PWLE commented:

“One of the things I really noticed is that I felt that the voices of people with lived and living experience was actually being valued. I have come to feel that some of that was tokenism, straight up, and some of it was genuine”. PWLE, Northern Region

Encouraging, motivating, and supporting other PWLE, feeling more in control and believing in ourselves were often intertwined. By feeling more confident and believing in themselves, PWLE often began to feel more in control and were increasingly able to encourage, motivate and support other PWLE. PWLE expressed repeatedly how meaningful their work was in harm reduction, overdose prevention and outreach. While it is essential that PWLE are paid equitably for their work and some PWLE were able to access housing, car ownership and other opportunities through paid employment, the most common incentive to engage in advocacy and community-based work was altruism and a desire to give back. Doing this work made PWLE feel like they belonged in their communities. Comments included:

“I’ve felt like I was someone that was not part of the community, or society, I wasn’t giving back. And now I actually feel like I’m part of the community, and I feel there are people that appreciate just a brown bag of lunch on Sunday. So yeah, that’s what it’s meant to me, it made me feel human again”. PWLE, Northern Region.

Between 2018 and 2021, CIE distributed over half a million dollars in grant funding to 32 PWLE groups in 20 different communities. This and other sources of funding were instrumental in supporting PWLE to establish and maintain supportive peer groups across the province. The purpose of these groups ranged from providing social support and a sense of community, to advocating for PWLE priorities, representing PWLE on committees and at decision-making tables, and providing outreach, harm reduction and community services. Being part of establishing and participating in a PWLE group was extremely impactful for many PWLE. It provided many PWLE with opportunities to develop leadership skills and access employment opportunities. The external analysis found that funding was instrumental to the successful formation and continuation of PWLE groups. PWLE commented:

“We were tenting it at one time as well, and between having a house, a home to live in, and the [PWLE] group, and …it’s just given us a sense of direction again and some confidence to go forward, and a giant help for people that are coming up behind us. If we do the hard work now, it won’t be so hard for them, right?” PWLE, interior region

“Now that we’ve had that space, we’re going on our fourth month now being open, seeing the light in [PWLE] eyes. They’re like ‘man, we needed this for so long, you folks have done amazing!’ the community has changed…you can see hope in their eyes, you can see self-worth in their eyes”. PWLE, Northern Region

Working with health authorities, community agencies and municipalities was often a positive experience for PWLE. There were also tensions when PWLE were simultaneously advocating for their priorities and trying to collaborate with partners who had different or opposing priorities. The external analysis revealed themes of disconnection between PWLE and some service providers at a health authority or municipal level, specifically when it came to decision-making by PWLE. There were power dynamics that continued to play out with health authority and agency staff and community leaders who were perceived to withhold support, tokenize PWLE, provide inconsistent support and sometimes be blatantly hostile and oppositional.

“And we don’t get the same voice, we don’t have the voice that’s amplified the same way. We’re in the work of social justice …I need to put quotations around progress or progressive because we’re not included in that. Where it is to a degree, but it’s a bunch of people from the outside who are making sure that you have enough women or enough people of each different ethnicity, but it’s not about experience. They don’t understand that this is a group of people that don’t have anything. And then people come from an academic background and try to use superficial differences to dictate a diverse group, but it does not take into account … voices from people on the ground who have actually been ignored while they’re standing in line at the soup kitchen. They’ve been through all of these things and they had to fight for whatever they had, it’s different work for somebody”. PWLE, Island Region.

### Service provider – progress markers

Service provider is an umbrella term that includes frontline harm reduction workers, managers, and senior leaders in regional health authorities, municipalities, and community-based organizations. PM two and seven appeared most frequently in the service provider responses. Several of the PM were present in clusters. PM one, eight and 22 appeared together as did PM 21, 27 and 29; and PM 15 and 30.

There were a wide range of responses that varied considerably from region to region depending on the starting point for PWLE inclusion and engagement when CIE began, the connections between the health authorities and community agencies, the commitment of individuals to the work and the support they received from leadership.

The PM that was coded most frequently was articulating key challenges and barriers to PWLE engagement. Including PWLE in service planning, provision and evaluation was a new concept in some regions in 2015. Champions of PWLE inclusion experienced several challenges. Within health authorities, service providers cited lack of support from leadership; lack of employment and payment standards for PWLE; lack of understanding of equity, trauma-informed practice and harm reduction principles among staff and colleagues; stigma against PWLE; rigid structures that were not adaptable; and lack of resources to address the overdose crisis as barriers to action. Within community agencies service providers cited lack of funding; high staff turnover; lack of capacity among staff; and community resistance. The external thematic analysis identified structural issues such as power inequalities between service providers and PWLE and a lack of funding for harm reduction services in the system. Chronic underfunding would often lead to staff burnout, understaffing and challenges with staff retention. These issues impacted PWLE as they would form trusting relationships with staff ‘champions’ but then have to continuously work with new staff or no longer work with staff who prioritized CIE work.

Identifying, supporting, and investing in allies and champions was also an important outcome of CIE for service providers. The capacity building and collaborative dialogue sessions provided opportunities for networking across agencies and participation in CIE served as an indicator of co-workers’ orientation toward equity and harm reduction. Those working in harm reduction felt a sense of community among others who shared some of their difficult and emotionally challenging experiences. One service provider commented:

“There's an emotionality to it, there's a gravity to this work that is not easily forgotten. And it only takes one person in the room to go, "Were you at that?" And then you could just see people percolate right up. It's legacy type work”. Service Provider, Island Region.

Service providers were able to identify allies in other sectors such as community agencies, municipal government, police, businesses, and legal advocacy. CIE created a loose-knit, informal community of practice that became a support network for innovators and harm reduction champions who often faced resistance in their communities and workplaces. Many harm reduction workers and champions felt burned out after five years of struggling to get attention and resources to address the overdose crisis. One service provider commented:

“I wonder as the overdose crisis continues and then whatever mental health crisis sort of implodes upon us post-COVID, that we feel as though we've been doing this for so long, maybe we don't need this anymore. I hope that none of that ever happens and that we recognize that we can't just become complacent. And dialogues and engagement sessions, sometimes even if they don't result in what might initially be perceived as a massive change, they may have provided a vitally important space to just feel connected or loved or heard, and I don't think that those should be… I think they need to be equally recognized for what they're worth. And we need to still create spaces where that safety can happen and where we can come together at a time that otherwise feels just quite overwhelming and often very alone”. Service Provider, Interior Region.

Working with PWLE to define processes, standards of practice and competencies for PWLE engagement and employment, staying connected to PWLE, and PWLE are at decision-making tables often appeared together. Those service providers that were able to create opportunities to work collaboratively with PWLE were able to maintain relationships and connections and often advocated for additional opportunities for PWLE. They identified many challenges with PWLE inclusion such as a lack of payment mechanisms and standards; expectations of other service providers; and difficulty maintaining personal and professional boundaries particularly when PWLE were also clients. The external analysis team found that organizational policies like PWLE payment guides and guidelines for working with PWLE emerged in communities where there has been long-term engagement with organized PWLE groups.

There was a wide range of support from health authorities for PWLE organizations, but where it was present, service providers were an active part of the co-ordination and integration of structured PWLE involvement. This was often facilitated by the presence of dedicated PWLE engagement staff and paid PWLE positions. Health authorities and community agencies had different challenges creating paid PWLE positions. Health authorities often had more rigid structures that made it difficult to create innovative and appropriate hiring and payment policies for PWLE. Community agencies often did not have enough funding to support paid PWLE positions. The external analysis identified the lack of structured funding for harm reduction positions, including PWLE, impeded PWLE from moving to a place of meaningful decision-making. The inherent power differential between health authorities who provided services and PWLE as former of present service users was often difficult to deconstruct within decision making and working relationships between PWLE and health authority staff.

CIE provided the opportunity for service providers to engage in critical self-reflection. Most service providers worked in very demanding, stressful, and fast paced environments that do not allow for moments of self-reflection on their work. Being given the time to reflect and connect with other harm reduction champions inspired many to recommit to modelling equity and inclusion in their daily work. CIE also provided a model for PWLE inclusion that service providers could use in other settings, particularly if they continued to collaborate with PWLE who had also attended CIE sessions. For example, the deliberate and thoughtful planning of the CIE gatherings fostered and strengthened relationships between PWLE and service providers. Intentional seating plans where service providers and PWLE were seated together and preparation with each group before collaborative dialogue sessions supported respectful communication and increased understand of each other’s perspectives. One service provider noted,

“It was more interactive, more focused, more of a focus on reducing the power differentials between everyone who was participating and… So that was good learning, in terms of modelling ways that we can be together”. Service Provider, Interior Region.

A PWLE participant echoed the theme,

“I found that having that big meeting and being at the tables with [PWLE] and service providers together at every different table was very helpful on that, 'cause we had those conversations together as a group. And they had an idea at every table that they were working with, so there were many different ideas that were being talked about at different tables, and we forged partnerships doing those meetings". PWLE, Northern Region.

Both service providers and PWLE commented on the persistence of stigma against PWLE, historical and ongoing PWLE experiences of trauma and Indigenous specific racism, affirming the ongoing need for interventions such as CIE.

Service providers observed:

“…there’s still so much stigma and blatant, like, lack of desire to understand and so people judge. And that further ostracizes people, you know, from, say, getting a job or volunteering or feeling like people do support and want the best for you. Because that’s not been their experience, right. So they’re just missed opportunities which I think happens far too often”. Service Provider, Northern Region, PWLE observed:

“There's a lot of stigma involved around this whole thing, which was making people die, I feel. I think the stigma is one of the biggest reasons that we don't know when to check on our loved ones. 'Cause when they're too scared to tell us that they're using, because of the stigma, and instead of checking on them, we're finding them dead”. PWLE, Interior Region.

“I know definitely, there's a big divide here between the Indigenous people and everybody else, which has gone on forever. That was no different when I grew up here”. PWLE, Northern region.

## Discussion and conclusions

CIE began with the intention of exerting influence on the health system to increase PWLE engagement where harm reduction services were newly established or not serving PWLE well. Through the process of developing the Progress Markers and successive adaptive planning cycles, it became clear that directly supporting PWLE facilitated more meaningful and lasting change than solely working to improve the health and social services that supported them. Opportunities for skills development, education, funding, and support to self-organize, and participation in decision-making processes contributed to PWLE’s wellbeing by increasing community involvement, strengthening social networks, and improving connections to social supports. The impacts of the CIE initiative extend far beyond the outcomes of any of the dialogues it facilitated and are largely the result of an increase in social capital.

Social capital is a concept that has informed public health for over 20 years [56]. It refers to the resources people have access to through their social networks and the relationships and shared values that connect groups and individuals, enabling them to work together. If CIE were to evaluate its success only by the number of measurable policy or practice changes that had resulted from its work, it would miss the more profound impacts it had through building and supporting the creation of social capital among and between PWLE and service providers. We may not have been attending to these important shifts if we had not collaborated with our boundary partners in defining the outcomes of the project from the outset.

Resistance to change and systemic inertia have had deadly consequences over the past six years. Both PWLE and service providers expressed frustration at the lack of action from health authorities amid rising death tolls. CIE engagements created the opportunity for change by inviting people most affected by the toxic drug supply together with those committed to supporting them, but their ability to bring about systemic change was limited. Both PWLE and service providers noted the lack of support to attend CIE engagements, lack of support for actions that came from CIE engagements, and lack of PWLE inclusion in decision-making by health authority leadership as limiting factors for systemic change. The lack of response at a systemic level often resulted in PWLE carrying the burden of responding to toxic drug poisonings. Commitment to their community and personal connections often compelled PWLE to act, often without the tools, resources and supports to do so and almost always without compensation.

While almost one-third of PWLE interviewed self-identified as Indigenous, none of the service providers interviewed for this evaluation identified as Indigenous. The lack of participation by Indigenous service providers may be indicative of a general lack of Indigenous service providers in the field of harm reduction. The low number of Indigenous healthcare workers in BC has been seen as symptomatic of the systemic racism and marginalization experienced by Indigenous Peoples more generally [57]. Shared experiences of colonization, racism, and an appreciation of divergent worldviews by Indigenous service providers could benefit Indigenous PWLE accessing harm reduction services. Just as PWLE can relate to and support other PWLE, Indigenous service providers could better support Indigenous PWLE.

The arrival of COVID-19 in 2020 had a devastating effect on PWLE in BC and made the work of CIE much more difficult. There are several causative pathways that have been proposed for the correlation between COVID-19 and increased overdose deaths, including stress, anxiety, despair, and worsening mental health as a result of social isolation and quarantine [58]. The Mental Health Commission of Canada reported that in November–December 2020, 30% of people with a substance use disorder diagnosis within their lifetime and 15% of people reporting current symptoms of problematic alcohol use had seriously contemplated suicide since the onset of the pandemic. During the same period, 5% of the general public had contemplated suicide [59].

Interventions like CIE could be effective at slowing the ongoing drug poisoning crisis, both during and after the most acute phases of the COVID-19 pandemic by fostering social connections and building social capital.

Integrating PWLE and Indigenous people into the planning, provision, and evaluation of harm reduction services will better serve those accessing services and provide valuable social connections and employment opportunities. Harm reduction and overdose prevention services must prioritize hiring, training, and retaining Indigenous people to provide culturally appropriate care and priority should be given to hiring, training, and mentoring Indigenous PWLE workers.

Service providers and PWLE who participated in CIE noted the need for consistent employment standards that recognize the work of PWLE and provide equitable compensation.

Even in the context of CIE dialogues, PWLE sometimes felt their unique knowledge and community connections were not always valued. Training and educational opportunities for service providers to understand their own biases towards PWLE and Indigenous people and the impacts they can have on care could increase social inclusion and help to address stigma.

### Limitations

This evaluation analysis has several limitations. First, only 10% of CIE participants participated in the evaluation and most were champions of the project. Peer participants’ interviews may have been influenced by receiving grant funding from CIE. No Indigenous service providers participated in the interviews. Finally, some of these interviews took place several years after CIE engagements so recall may be limited.

## Data Availability

The datasets generated and/or analysed during the current study are not publicly available to protect the privacy of participants but are available from the corresponding author on reasonable request.

## Abbreviations

BC: British Columbia
BCCDC: British Columbia Centre for Disease Control
CIE: Compassion, Inclusion and Engagement initiative
FNHA: First Nations Health Authority
OM: Outcome mapping
PM: Progress markers
PWLE: People with lived experience of substance use
